# Controllable Synthesis of Copper Oxide/Carbon Core/Shell Nanowire Arrays and Their Application for Electrochemical Energy Storage

**DOI:** 10.3390/nano5041610

**Published:** 2015-10-09

**Authors:** Jiye Zhan, Minghua Chen, Xinhui Xia

**Affiliations:** 1 State Key Laboratory of Silicon Materials, Key Laboratory of Advanced Materials and Applications for Batteries of Zhejiang Province, and School of Materials Science and Engineering, Zhejiang University, Hangzhou 310027, China; E-Mail: zhanjiye@yeah.net; 2 School of Applied Science, Harbin University of Science and Technology, Harbin 150080, China

**Keywords:** core/shell structure, anode, nanowire arrays, copper oxides, lithium ion batteries

## Abstract

Rational design/fabrication of integrated porous metal oxide arrays is critical for the construction of advanced electrochemical devices. Herein, we report self-supported CuO/C core/shell nanowire arrays prepared by the combination of electro-deposition and chemical vapor deposition methods. CuO/C nanowires with diameters of ~400 nm grow quasi-vertically to the substrates forming three-dimensional arrays architecture. A thin carbon shell is uniformly coated on the CuO nanowire cores. As an anode of lithium ion batteries, the resultant CuO/C nanowire arrays are demonstrated to have high specific capacity (672 mAh·g^−1^ at 0.2 C) and good cycle stability (425 mAh·g^−1^ at 1 C up to 150 cycles). The core/shell arrays structure plays positive roles in the enhancement of Li ion storage due to fast ion/electron transfer path, good strain accommodation and sufficient contact between electrolyte and active materials.

## 1. Introduction

The pursuit of better performing, longer lasting lithium ion batteries (LIBs) is driven by the rising demand of battery-powered devices, vehicles and machinery [1,2,3]. It is well known that the performance of LIBs is mainly controlled by the properties of active electrode materials, which are influenced by their physicochemical properties (e.g., morphology, crystal and composition). In order to increase the energy output and reduce the battery weight, exploring anode materials with high specific capacities to substitute the current commercial graphite is an attractive and indispensable route [4]. To date, transition metal oxides have been widely studied as promising candidates because of their high specific capacity, typically 2–3 times higher than that of the commercial graphite/carbon based materials [5,6,7,8,9]. Among them, copper oxide (CuO) has attracted increasing attention due to its high theoretical capacity (674 mAh·g^−1^) [10], arising from reversible electrochemical reactions with Li ion (CuO + 2Li^+^ + 2e^−^ → Cu + Li_2_O). However, similar to other metal oxides, the practical application of CuO as anode for LIBs is still hindered by two main issues: pulverization problem and fast capacity fading due to the aggregation, large volume changes and severe destruction of the electrode. To overcome these problems, in recent years, comprehensive arrays electrode design has been developed to boost the power/energy densities and improve cycling stability. Compared with the traditional powder materials, the integrated nanoarrays electrodes show several advantages. First, individual active arrays grow directly on the conductive substrates and ensure good electric connection with the current collectors. Second, there is no need to add additional polymer binders and additives to the electrode and avoid undesirable supplementary interfaces leading to lower internal resistance [11]. Third, fast ion/electrons transfer path are provided by the open porous arrays architecture, resulting in enhanced reaction kinetics and higher utilization of active materials [12,13]. Fourth, better accommodation of large volume changes and the structural stress and lead to improved cycling stability. This integrated electrode design has proven to be successful in metal oxide systems such as NiO [12], Fe_3_O_4_ [14,15], Co_3_O_4_ [16], SnO_2_ [17], and CuO [18]. Currently, a few integrated CuO nanostructured electrodes (e.g., nanosheets, and nanowires) have been reported, and enhanced results have been demonstrated in the CuO-based materials. In particular, 1D nanowire structures arouse considerable research interest due to their rich accessible electro-active sites and fast lithium ion diffusion [19].

To date, 1D CuO nanowire arrays have been prepared by oxidation and anodic electro-deposition methods, respectively. Zhang *et al.*, reported 1D CuO nanowire arrays prepared by anodic electro-deposition method and their lithium ion storage properties [20]. However, the pure CuO nanowire arrays are still not satisfactory because of their relatively low electrical conductivity. Recently, researchers have adopted an outer conductive layer (such as graphene quantum dot) to further improve the electrical conductivity of CuO nanowires and structural stability. Fan group coated graphene quantum dots on the surface of CuO nanowires to obtain enhanced performance of LIBs [21], but the distribution of graphene quantum dots layer is not homogeneous.

Inspired by these exciting results, in this work, we report porous CuO/C core/shell arrays directly on nickel foam by a combination of electrodeposited and chemical vapor deposition method. CuO/C core/shell nanowires with diameters of ~400 nm are formed and grow quasi-vertically to the substrate. Highly porous structure and good electrical conductivity is combined in one electrode. As an integrated anode for LIBs, the as-prepared CuO/C nanowire arrays exhibit high discharge capacity and noticeable high-rate capability due to the unique porous array structure with fast ion/electron transfer and large contact area between active materials and electrolyte. The proposed method can be applicable for preparation of other high-performance hierarchical porous metal oxide arrays for applications in sensors, catalysis and energy storage and conversion.

## 2. Results and Discussion

**Figure 1:**
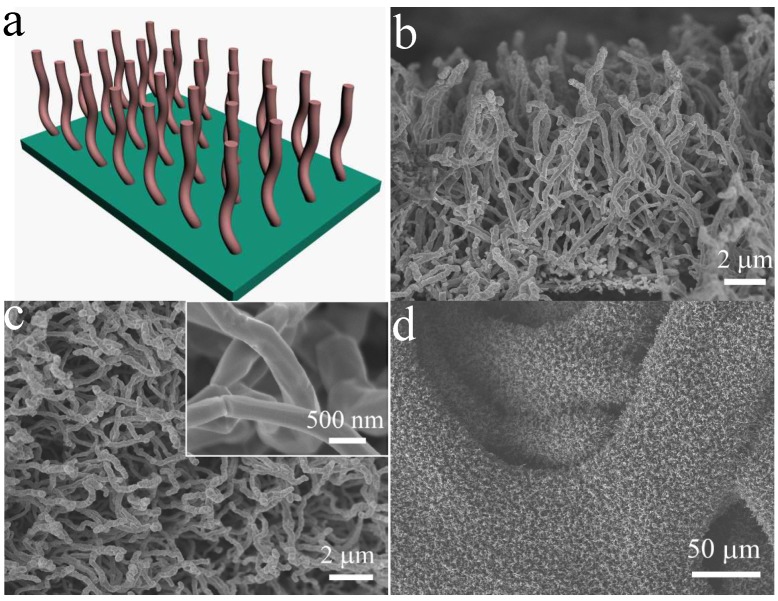
(**a**) Schematics of growth of CuO/C core/shell nanowire arrays; (**b**–**d**) Scanning electron microscopy (SEM) images of CuO/C core/shell nanowire arrays (inset: high-magnification SEM image).

**Figure 2:**
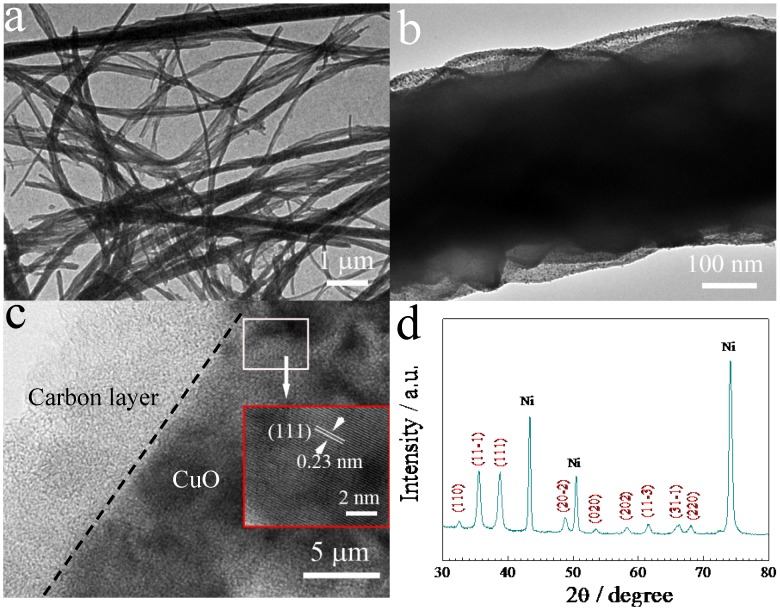

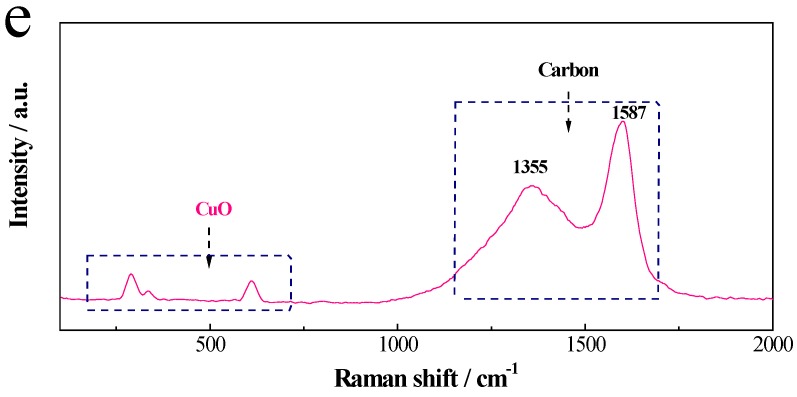
(**a**–**c**) Transmission electron microscopy (TEM) images of CuO/C core/shell nanowires (HRTEM image in inset); (**d**) X-ray power diffraction (XRD) pattern of CuO/C core/shell nanowires; (**e**) Raman spectrum of CuO/C core/shell nanowires.

Figure 1a shows the schematics of growth of CuO/C nanowire arrays, confirmed by the cross-sectional scanning electron microscopy (SEM) image (Figure 1b). It can clearly be seen that the CuO/C core/shell nanowires grow quasi-vertically to the substrate forming 3D arrays. Note that each nanowire has its own contact with the substrate at the bottom. This can ensure every nanowire to participate in the electrochemical reaction. As shown in Figure 1c,d, the nickel foam substrate is homogeneously covered by the CuO/C nanowires with diameters of ~400 nm. The surface of nanowires is smooth, not rough. There is lots of open space between neighboring nanowires. This characteristic allows for easy diffusion of electrolyte into the inner region of the electrode, and will be beneficial for the reduction of internal resistance and improvement of high-power performance. The core/shell structure of the CuO/C nanowires is confirmed by the transmission electron microscopy (TEM) results. Figure 2a–c shows the TEM images of CuO/C nanowires and verifies that the diameters of core/shell nanowires are about 400 nm, and a thin amorphous carbon shell of ~10 nm is uniformly coated on the surface of CuO nanowires. HRTEM image in the inset shows well-resolved atomic lattice fringes with inter-planar spacing of 0.23 nm, consistent with the value of the (111) plane of cubic CuO. The crystal structures of samples are analyzed by XRD analysis (Figure 2d). Except for the peaks from nickel foil substrate, the other peaks are characteristic of CuO (JCPDS 80-1917), no diffraction peaks of carbon are noticed, implying its amorphous nature, supported by the Raman test (Figure 2e). The Raman spectrum clearly shows two obvious peaks located at 1355 cm^−1^ (D-band) and 1587 cm^−1^ (G-band), which are characteristic of amorphous carbon (Figure 2e). The other peaks (288 cm^−1^, 335 cm^−1^ and 611 cm^−1^) in the low wavelength region are characteristic of CuO phase. All the results above are in agreement with each other, indicating the formation of CuO/C core/shell arrays.

**Figure 3:**
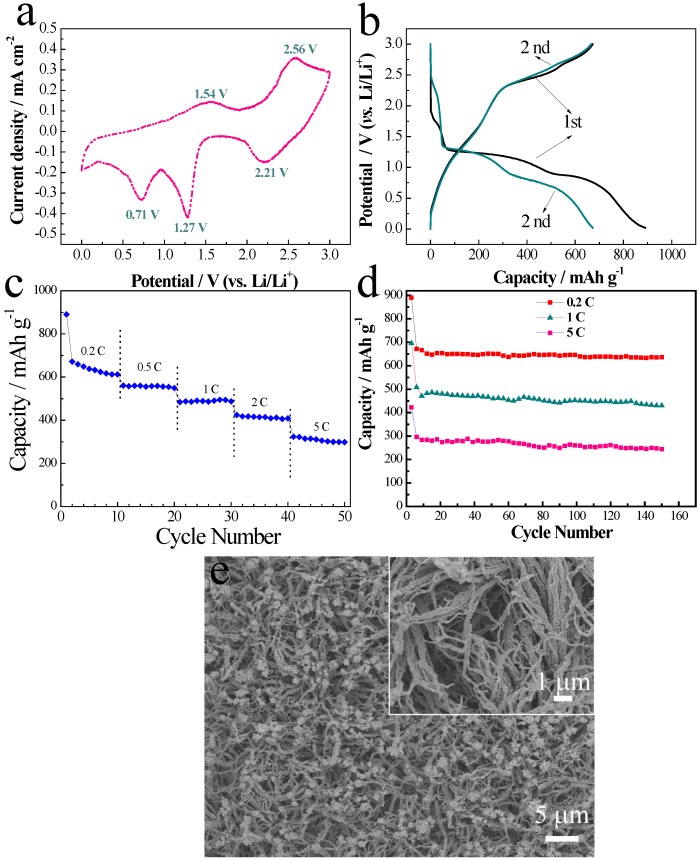
Electrochemical characterizations of CuO/C core/shell nanowires as anode of lithium ion batteries (LIBs). (**a**) Cyclic voltammetry (CV) curve at a scan of 0.1 V·s^−1^ at the second cycle; (**b**) First charge/discharge profiles at 0.2 C; (**c**) Rate capability; (**d**) Cycling life at different rates; (**e**) SEM morphology after 150 cycles at 0.2 C.

The electrochemical properties of the obtained CuO/C core/shell arrays are characterized as anode of LIBs. CV curve of the CuO/C core/shell arrays at the second cycle is shown in Figure 3a.

During the cathodic process, three cathodic peaks around 2.21, 1.27 and 0.71 V are observed, and correspond to C multi-step electrochemical lithium reaction processes related to the formation of intermediate Cu_1−_*_x_*CuxO_1−_*_x_*_/2_ solid solution, further reduction to Cu_2_O, and conversion to Cu and Li_2_O, as well as the formation of solid electrolyte interface (SEI) film, respectively [22]. In the anodic process, two peaks at 1.54 and 2.56 V are due to the partial decomposition of SEI film and reformation of CuO. Figure 3b shows the first discharge/charge curves of CuO/C core/shell nanowire arrays at 0.2 C. The charge/discharge plateaus are consistent with those observed in cyclic voltammetry (CV) curve. The first discharge and charge capacity of the CuO/C core/shell nanowire is 890 and 672 mAh·g^−1^ respectively, with an initial columbic efficiency of 75.5%. The capacity loss at the first cycle is mainly due to irreversible formation of the SEI film and the decomposition of electrolyte [23], which happens in all 3D transition mental oxides, including NiO [24], Co_3_O4 [16], Fe_3_O_4_ [25], SnO_2_ [26], and Fe_2_O_3_ [27]. The additional capacity of conversion reaction anodes is mainly based on some possible reason: (i) reversible decomposition of the electrolyte as a side reaction associated with the formation of a solid electrolyte interface (SEI) layer; and (ii) extra Li^+^ adsorption-desorption on the SEI (interfacial storage) [28,29]. In addition, Hu and co-workers found that the additional capacity at the first discharge cycle might come from reversible SEI formation, and the formation of LiOH and its subsequent reversible conversion to Li_2_O and LiH [30].

Specific capacity of the CuO/C core/shell nanowire arrays at different discharge current densities are shown in Figure 3c. The CuO/C core/shell nanowire arrays show good high-rate reversible capability of 672 mAh·g^−1^ at 0.2 C, 555 mAh·g^−1^ at 0.5 C, 486 mAh·g^−1^ at 1 C, 420 mAh·g^−1^ at 2 C, and 320 mAh·g^−1^ at 5 C, respectively. A capacity retention of 47.6% is obtained when current density increases from 0.2 C to 5 C. In view of low capacity and low load mass of C in the composite arrays, the capacity contribution from the C is very small, lower than 2%. The actual capacity of CuO is a litter higher than the current value. Take the value at 0.5 C for example, the CuO/C shows a specific capacity of 555 mAh·g^−1^ at 0.5 C. After considering the different capacity contribution, the pure CuO nanowires have a capacity of about 565 mAh·g^−1^ at 0.5 C. Electrochemical cycling performances of the CuO/C nanowire arrays electrode at different rates are illustrated in Figure 3d. Observe that the CuO/C core/shell nanowire arrays exhibit good cycling stability. After 150 cycles, a reversible capacity of 633 mAh·g^−1^ at 0.2 C, 432 mAh·g^−1^ at 1 C, and 237 mAh·g^−1^ at 5 C, is obtained, respectively. The results are higher than the pure electrodeposited CuO nanowires [20], and comparable to carbon flakes decorated CuO nanowires [31].

The good performance of the CuO/C core/shell nanowire arrays is mainly due to the following positive aspects. (1) Direct growth of CuO/C nanowire arrays result in good physical and electrical contact between the active materials and current collector [24,31]; (2) The open nanowire structure facilitates electrolyte penetration to every part of the film and thus shortens the diffusion paths for both electrons and lithium ions within the oxides [31]; (3) The overall porous structure provides larger reaction surface and inner space favoring the efficient contact between active materials and electrolyte. This feature provides more active sites for electrochemical reactions and allows facile lithium ion diffusion at high current density; (4) The core/shell architecture possesses good mechanical stability. After 150 cycles at 0.2 C, the entire structure of the CuO/C nanowire arrays is well maintained (Figure 3e). The enhanced mechanical stability is favorable for obtaining better cycling life.

## 3. Experimental Section

The CuO nanowire arrays were prepared by a facile anodic electro-deposition (ED) method. First, copper was deposited on the nickel foam by a two-electrode cathodic ED method. The electrolyte consisted of 1 M Cu(NO_3_)_2_ + 0.5 M NaNO_3_. The deposition of Cu was conducted at 1 mA·cm^−2^ for 10 min. After that, the samples was followed by another ED, which was performed in a standard three-electrode glass cell at 25 °C, the Cu on nickel foam as the working electrode, Hg/HgO electrodes the reference electrode and a Pt foil as the counter-electrode. The electrolyte was 1 M KOK. The anodic electro-deposition experiments were carried out at constant anodic current of 10 mA·cm^−2^ for 2700 s. Finally, the as-prepared samples were rinsed and then annealed at 450 °C for 2 h in flowing argon. Then the samples were followed by a chemical vapor deposition (CVD) to form CuO/C core/shell arrays. The carbon source was ethanol. The CVD was conducted at 600 °C for 5 min in mixed flowing H_2_ (5%) + Ar. The ethanol was bubbled through into the tube by the H_2_ (5%) + Ar gas (150 sccm). The load weight of CuO was about 2.5 mg·cm^−2^ and the carbon accounted for about 5 wt % in the composite. In our experiment, the sample we weighted by using an analytical balance with high measure resolution (0.01 mg). First, we calculated the load weight of CuO by the mass difference before and after the preparation of CuO nanowire arrays on the substrate. Then, we calculated the load weight of CuO/C by the mass difference before and after the loading of CuO/C on substrate. To check the mass of carbon and CuO, we dissolved the sample by 0.5 M HCl aqueous solution. Then, inductively coupled plasma-optical emission spectroscopy (ICP-OES, Spectro Arcos, SPECTRO Analytical Instruments GmbH, Kleve, Germany) analysis was used to determine the precise composition of Cu in the solution. Besides, elemental analysis was also used to determine the mass of carbon. Combing these methods, we got the final load weight of different components.

The morphology and microstructure of the sample were characterized by a scanning electron microscopy (SEM, Hitachi S-4700 and FESEM, FEI Sirion-100, U-PICA Co., Ltd., Tokyo, Japan), transmission electron microscopy (TEM, JEM 200CX at 160 kV, Tecnai G2 F30 at 300 kV, Japan Electronics Co., Ltd., Tokyo, Japan), Raman spectroscopy (WITec-CRM200 Raman system with a laser wavelength of 532 nm, WITec Wissenschaftliche Instrumente und Technologie GmbH, Ulm, Germany), and X-ray power diffraction (XRD, Rigaku D/max 2550 PC, Cu Kα, Rigaku Corporation, Tokyo, Japan).

The electrochemical tests were carried out using a coin-type half cell (CR 2025) with pure lithium foil as both the counter and the reference electrodes. The CuO/C nanowire arrays were directly used as the working electrode. Test cells were assembled in an Ar-filled glove box. The electrolyte was 1 M LiPF_6_ in ethylene carbonate (EC)-dimethyl carbonate (DME) (1:1 in volume), a polypropylene (PP) micro-porous film (Cellgard 2300) as the separator. The galvanostatic charge-discharge tests were conducted on a LAND battery program-control test system at room temperature (25 ± 1 °C). Cyclic voltammetry curves were scanned at 0.1 mV·s^−1^ using an electrochemistry system (CHI 660E).

## 4. Conclusions

In summary, we have demonstrated a simple and versatile method to fabricate CuO/C core/shell nanowire arrays. In such rational designed structure, both high electrochemical capacity and improved cyclic stability are achieved. As the anode for LIBs, the CuO/C core/shell nanowire arrays exhibit a high discharge capacity of 633 mAh·g^−1^ at 0.2 C after 150 cycles. We are optimistic that the developed method can be extended to prepare other core/shell arrays for applications in supercapacitors, full cells and LIBs.
